# CNT-based saturable absorbers with scalable modulation depth for Thulium-doped fiber lasers operating at 1.9 μm

**DOI:** 10.1038/srep45491

**Published:** 2017-04-03

**Authors:** Grzegorz Sobon, Anna Duzynska, Michał Świniarski, Jarosław Judek, Jarosław Sotor, Mariusz Zdrojek

**Affiliations:** 1 Laser & Fiber Electronics Group, Faculty of Electronics, Wroclaw University of Science and Technology, Wybrzeze Wyspianskiego 27, 50-370 Wroclaw, Poland; 2 Faculty of Physics, Warsaw University of Technology, Koszykowa 75, 00-662 Warsaw, Poland

## Abstract

In this work, we demonstrate a comprehensive study on the nonlinear parameters of carbon nanotube (CNT) saturable absorbers (SA) as a function of the nanotube film thickness. We have fabricated a set of four saturable absorbers with different CNT thickness, ranging from 50 to 200 nm. The CNTs were fabricated via a vacuum filtration technique and deposited on fiber connector end facets. Each SA was characterized in terms of nonlinear transmittance (i.e. optical modulation depth) and tested in a Thulium-doped fiber laser. We show, that increasing the thickness of the CNT layer significantly increases the modulation depth (up to 17.3% with 200 nm thick layer), which strongly influences the central wavelength of the laser, but moderately affects the pulse duration. It means, that choosing the SA with defined CNT thickness might be an efficient method for wavelength-tuning of the laser, without degrading the pulse duration. In our setup, the best performance in terms of bandwidth and pulse duration (8.5 nm and 501 fs, respectively) were obtained with 100 nm thick CNT layer. This is also, to our knowledge, the first demonstration of a fully polarization-maintaining mode-locked Tm-doped laser based on CNT saturable absorber.

Fiber lasers operating in the 1.9–2.0 micron range were one of the most intensively developed topics of laser technology over the past decade. Especially Thulium-doped fiber lasers (TDFLs) have experienced tremendous advancement. Robust and compact TDFLs might find many practical applications, like medical procedures (e.g. ablation of urinary tissues^1^,^2^, cellular-precision surgery^3^), or laser absorption spectroscopy^4^. The spectrum generated by TDFLs overlaps with absorption lines of several molecules, e.g. nitrous oxide (N_2_O), carbon dioxide (CO_2_), or hydrogen bromide (HBr), which opens the possibility of developing cost-effective trace-gas sensing platforms. Ultrashort-pulsed TDFLs are also particularly desirable in nonlinear optics and mid-infrared generation. As an example, mode-locked TDFLs can serve as pump sources for optical parametric oscillators^5^ or supercontinuum generation in highly nonlinear (e.g. photonic crystal) fibers^6^,^7^.

Ultrafast Thulium-doped fiber lasers might be mode-locked using variety of techniques. The most popular include: semiconductor saturable absorber mirrors (SESAMs)^8^, carbon nanotubes^9^,^10^, graphene^11^,^12^, topological insulators^13^, black phosphorus^14^, and nonlinear optical/amplifying loop mirrors (NOLM/NALM)^15^. All those mode-locking techniques are well-established and widely used by the laser community. The CNTs are very promising for 2 micron applications, due to their fast recovery time, broad operation range, low saturation intensity, and relatively low cost and easy fabrication^16^,^17^,^18^. They can also support very short pulse generation, up to 66 fs^19^, and are characterized by an optical damage threshold high enough to use them in typical fiber oscillators^20^. Despite those advantages, to date, there were only a very few reports on CNT-based Tm-doped mode-locked oscillators in the literature. The first mode-locked TDFL utilizing CNTs was demonstrated in 2008 by M. Solodyankin *et al*.^9^. The laser generated 2.05 ps-long pulses and used CNTs obtained via arc discharge, immersed in carboxymetyl cellulose polymer. One year later K. Kieu et al. reported a linear Tm-doped fiber laser with a SWCNT/polymer composite, generating 750 fs pulses^10^. The shortest pulses at 2 microns from CNT-based laser were reported very recently by J. Wang *et al*.^21^. Proper dispersion management of the cavity using normal dispersion fibers, as well as external compression led to generation of 152 fs-short pulses. Recently, a method of hybrid mode-locking of Tm-doped fiber lasers was proposed, where the oscillators contains a CNT saturable absorber, but simultaneously incorporates another mode-locking mechanism (e.g. NOLM^22^,^23^ or NPE^24^). Nevertheless, all previous reports on 2 micron mode-locked lasers utilizing CNTs were related to non-polarization maintaining cavities. Thus, in all those cases, the oscillators required the usage of a polarization controller (PC) in order to initiate or optimize the mode-locking and pulsed operation. In general, non-PM lasers tend to be unstable and vulnerable to external disturbances. Additionally, the polarization state of the output radiation is undetermined, which might be a serious drawback in case of many practical applications, which require linearly polarized beams.

Moreover, despite the number of reports on CNT-based Er- and Tm- lasers, there were no comprehensive studies carried out on controlling the nonlinear optical parameters (i.e. scaling modulation depth) of a CNT-SA. In all of the mentioned reports published so far, the laser performance was investigated for a specific, arbitrarily chosen nanotube layer thickness in the SA. It is usually also unknown why this specific thickness is chosen. The influence of the CNT layer thickness on the modulation depth and on the Tm-laser behavior was not investigated yet. In our previous works, we have examined the possibility of modulation depth scaling in graphene saturable absorbers^25^. We have revealed, that the number of graphene layers determines the modulation depth, and has a significant influence on a fiber laser behavior. In this work, we have fabricated a set of CNT-based saturable absorbers with four different layer thicknesses (50, 100, 150 and 200 nm). The CNTs were fabricated via a vacuum filtration method^26^,^27^ and deposited on fiber connectors. This method allows to fabricate a densely packed film, where CNTs are agglomerated forming closely packed carpet without any additional binder like polymer, in contrast to previous papers on CNT-based SAs^28^,^29^. However, previous studies have shown, that agglomeration of the nanotubes might cause an increase in the non-saturable losses of the SA^30^. One can avoid the agglomeration by mixing CNTs with graphene, as demonstrated in ref. 30. Nevertheless, our goal was to fabricate a polymer-free CNT film, which also can improve the damage threshold. As shown recently by S. Kobtsev et al.^31^, application of polymer-free CNT films solves the problems related to degradation of conventional polymer matrices of CNT-based SAs, and opens the possibility of fabricating longer-lasting and more reliable saturable absorbers. Another way of increasing of the damage threshold of the SA is to utilize the evanescent field interaction effect, i.e. deposit the material on a side-polished (D-shaped) fiber or a tapered microfiber. This approach was already investigated with CNTs^32^, as well as with other two-dimensional SA materials (e.g. graphene^33^, topological insulators^34^, and transition-metal dichalcogenides, like MoS_2_^35^).

Our technique presented here allows to fabricate clean, polymer-free CNT films with controllable thickness. Those films can be afterwards transferred onto fiber connectors, forming ideal saturable absorbers for all-fiber lasers developed in PM technology. We have studied the influence of the CNT film thickness on the nonlinear optical parameters (modulation depth, saturation fluence). Afterwards, the CNT-SAs were tested in a state-of-the-art, all polarization maintaining Tm-doped fiber laser. Such experimental study was not reported elsewhere in the literature so far. Our results show, that the broadest output spectrum (8.5 nm) and shortest pulses (501 fs) are generated using a 100 nm thick CNT layer in the SA. This is, to our knowledge, the first reported comprehensive study on controlling of the parameters of a SA by scaling the CNT layer thickness. We have also presented, to the best of our knowledge, the first fully polarization maintaining, mode-locked Tm-doped all-fiber laser with CNT saturable absorber. The PM design of the laser is extremely important and very advantageous in comparison to non-PM lasers. In a non-PM cavity, a polarization controller is necessary to adjust the polarization state inside the resonator and either initiate the pulsed operation^24^, optimize the mode-locking performance^36^, or ensure stable single-pulse operation^23^. Generally, non-PM lasers tend to be sensitive to external perturbations (like vibrations, movement of the fibers, temperature changes, etc.). Additionally, the output pulses from a non-PM cavity are in fact vector solitons, containing two different polarizations, that might freely evolve. The energy exchange between those two vector solitons may cause formation of sub-sidebands in the optical spectrum^37^,^38^. The only way to provide self-starting, turn-key and environmentally stable operation, is to develop the cavity in all-fiber technology, using PM fibers and components. Such lasers emit scalar solitons with one polarization state, which is crucial for many applications. Additionally, PM lasers are self-starting and possess only one fundamental mode-locking state (the mode-locking cannot be optimized in any way; the laser is completely alignment-free and the mode-locking performance cannot be affected/degraded by external factors).

## Results

**Figure 1:**
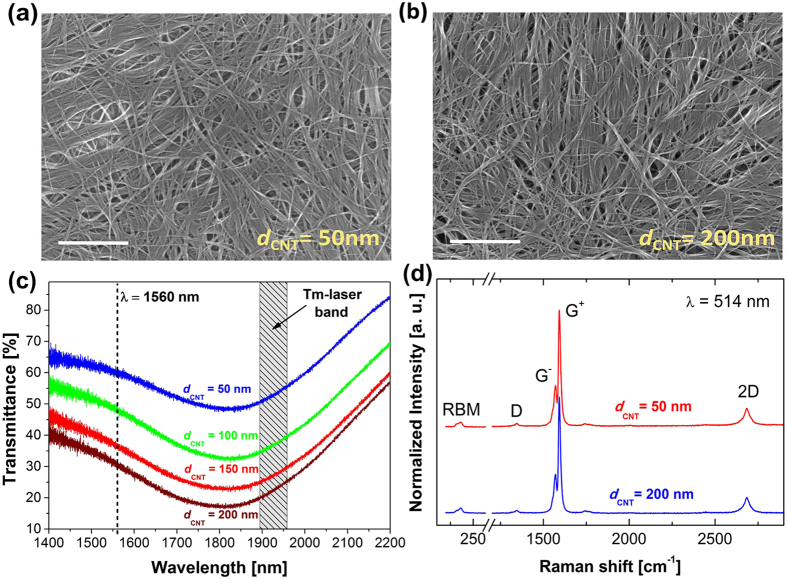
SEM images (**a**) and (**b**) of the CNT films with thicknesses 50 and 200 nm, respectively (scale bar represents 450 nm), (**c**) linear transmittance spectra of the films (**d**) Raman spectra of the CNT films (collected with 514 nm incident light). For SEM and Raman measurements, CNT films were transferred on the Si/SiO_2_ substrate, and on soda-lime glass for transmittance measurements.

As a starting point of the CNT film characterization (before deposition on the fiber connector) scanning electron microscope (SEM) imagining has been employed. The SEM images of the carbon nanotube films (on flat substrate) with thicknesses 50 and 200 nm are shown in Fig. 1a and b. We note that the vacuum filtered films are clean without residual contaminations and have similar tightly packed nanotube arrangement independent of the sample thickness. Figure 1(c) shows linear transmittance spectra for all studied CNT films in the range of 1400–2200 nm (films deposited on a glass substrate), with the Tm-doped laser emission range marked with gray color. For the wavelength of λ = 1560 nm (see the vertical black dotted line in the picture 1(c)), the transmittance level is approximately 60%, 48%, 36% and 30% for the CNT film with thickness 50, 100, 150 and 200 nm, respectively. Raman spectra in Fig. 1(d) exhibit typical carbon nanotube modes (RBM, G and 2D)^39^. Relatively small D peak confirms that fabricated films are clean and contain few defects.

**Figure 2:**
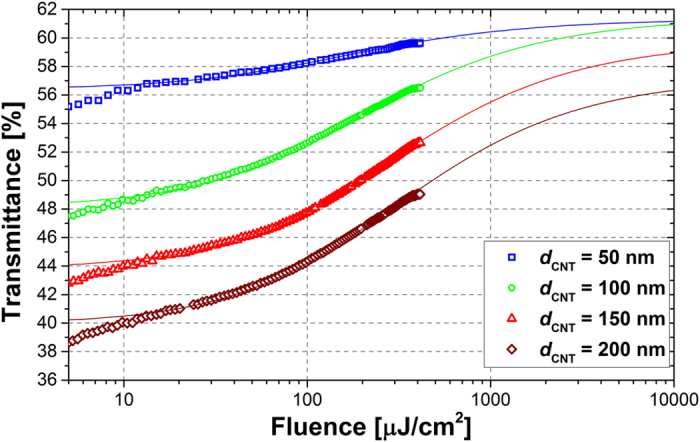
Measured power-dependent transmittance of the fabricated CNT samples together with calculated fitting curves.

The measured saturable absorption curves of all prepared samples (CNT films deposited on the fiber connectors), together with theoretical fitting (see “Methods”) are plotted in Fig. 2. All measurements were performed at 1560 nm wavelength. It can be seen, that the modulation depth scales with the thickness of the CNT layer. The measured modulation depth and saturation fluence values, together with the values used for the best possible fitting, are summarized in Table 1. The *F*_*sat*_ values were determined from the measured curves, as a point where the transmission increases by 1/e factor of the modulation depth^40^. Due to the fact, that in all cases we do not reach the theoretical modulation depth, the *F*_*sat*_ values read from the graph are approximately two times smaller than the calculated. Also the “real” modulation depth (*ΔT*), which could be measured in our setup, is approx. twice smaller than the value from the fit, due to insufficient power level to fully saturate the samples. Nevertheless, the obtained parameters are comparable to those reported in the literature for other CNT-SAs. For example, Z. Yu et al.^19^ reported 7.8% modulation depth, but the CNT layer was deposited on a tapered microfiber. The Authors in ref. 21 report 10% of modulation depth, but also in their case the sample was not fully saturated due to not enough pumping power. Modulation depth of 18% was reported in ref. 41, using a 30 μm-thick CNT film. It can be seen, that the transmittance levels of the fabricated CNT-SAs shown in Fig. 2 slightly differ from those presented in Fig. 1(c). It is caused most likely due to the additional attenuation of the fiber adapters used to join two connectors, or also due to the possibility of CNT film local folding during the sample preparation (transmittance characteristics from Fig. 1(c) were measured with CNTs deposited on glass substrates).

Due to lack of a proper power-dependent transmission setup at 2 μm, the nonlinear parameters were measured at 1560 nm. However, still this measurement gives us some information about the scalability of the modulation depth by changing the thickness of the CNT film, which was not studied so far. To the best of our knowledge, the dependence of the CNT nonlinear parameters on the wavelength was not investigated by any group yet. This is why it is extremely difficult to discuss how the wavelength might affect the accuracy of the nonlinear parameters. Nevertheless, it has been already show, that properly fabricated CNT-SAs might support mode-locking in solid-state lasers in a very broad range of wavelengths (1.07–1.95 μm)^42^,^43^.

### Setup of the PM Tm-doped laser

**Figure 3:**
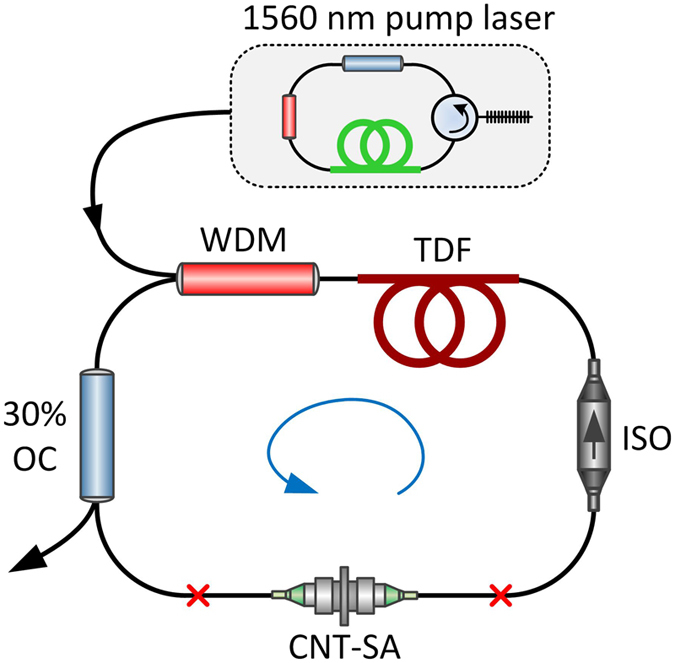
Setup of the all-fiber polarization maintaining Tm-doped oscillator.

The influence of the CNT-SA parameters on the behavior of the fiber laser was investigated in a fully fiberized, all polarization maintaining Tm-doped fiber oscillator. A schematic of the laser is depicted in Fig. 3. The resonator comprises: 12 cm long piece of Thulium-doped fiber (Nufern PM-TSF-5/125, TDF), a wavelength division multiplexer (WDM), an isolator (ISO), an 30% output coupler (OC), and the saturable absorber. The laser was counter-directionally pumped by a self-made 1560 nm fiber laser with maximum available power of 620 mW. The dispersion of the cavity was all-anomalous, and no dispersion compensation mechanisms (e.g. normal dispersion fibers) were used. All fibers and components used in the cavity were polarization maintaining – such design ensures stable, self-starting mode-locked operation, invulnerable to external disturbances, and provides exactly the same conditions for each tested saturable absorbers.

### Laser performance

In our experiments, all four tested saturable absorbers were spliced one after the other into the cavity. We have carefully controlled the lengths of the fibers, in order to maintain the repetition frequency of all setups. The maximum difference in repetition frequency between the lasers was 741 kHz, which corresponds to a fiber length difference of 4.7 cm (about 1.3% mismatch of the overall resonator length). We believe that such small mismatch is negligible and has no influence on the laser behavior, since the dispersion of the oscillator is fully anomalous. We have recorded 9 basic parameters of the laser: the threshold pump power required for stable mode-locking (P_pump_thr_), maximum pump power with stable mode-locking without any parasitic continuous-wave (CW) lasing (P_pump_max_), the central emission wavelength at maximum pump (λ_center_), the half width at half maximum bandwidth of the spectrum (Δλ_FWHM_), the pulse duration (τ_pulse_), the average output power (P_out_), the calculated pulse energy (E_p_), the time-bandwidth product (TBP), and the repetition frequency (f_rep_). All parameters are summarized in the Table 2.

The best performance in terms of bandwidth and pulse duration (8.5 nm and 501 fs, respectively) was obtained with 100 nm thick CNT layer. However, the differences in the pulse durations between the setups are not so significant as in case of graphene-based saturable absorbers with different number of layers^25^. The Tm-doped laser is quite sensitive to additional losses inside the cavity, which results in significant shift in the central wavelength (from 1925 nm to 1945 nm). However, bandwidths and pulse durations are very similar in all cases. It indicates, that choosing the SA with proper CNT thickness might be an efficient method for wavelength-tuning of the laser, without degrading the pulse duration, bandwidth, or output power. It can be seen, that in contrast to our previous laser based on graphene^25^, here the wavelength shifts towards longer wavelengths with increased cavity loss (caused by a thicker CNT-layer SA). The reason of that behavior is, that in case of this particular resonator, the optimum balance between the resonator transmittance, and the emission/absorption cross-sections of the active fiber is found at longer wavelengths, when the additional losses of the CNT-SA are introduced. We believe, that the emission wavelength of the laser is mostly determined by the transmittance characteristic of the saturable absorber. As it can be seen from the broadband characteristics shown in Fig. 1(c), the transmittance of the CNTs is strongly wavelength-dependent and increases at longer wavelengths (>1850 nm). In consequence, when a thicker SA is inserted to the cavity (e.g. the 200 nm sample with higher overall loss), the laser will compensate the losses and shift its emission towards longer wavelengths, where the SA exhibits higher transmittance. This behavior is much different than in case of graphene, since graphene has a wavelength-independent transmittance profile, i.e., the transmittance characteristic is quite flat over broad range of wavelengths. Thus, the emission wavelength of a graphene-based laser is not affected by the saturable absorber’s transmittance, but by other factors (like the gain profile of the active fiber). For the same reasons the output power increases with thicker CNT layers: at longer wavelengths, the absorption of the Tm-fiber drops significantly, and the transmittance of the SA increases. So most likely the wavelength shift induces better conversion efficiency and results in higher output power. We have also found, that the pump power threshold is the highest for the thinnest sample, and is lower for thicker samples. It can be explained by the value of the modulation depth required for mode-locking. Generally, soliton lasers operating in anomalous dispersion (like ours) requires relatively low modulation depth SA to initiate the pulsed operation (at the level of few percent). The required modulation threshold is achieved faster (i.e. at lower pump powers) for the thicker SAs, which have a much larger modulation depth in overall than the thinnest sample. In general, the output powers are much higher in the CNT-based lasers, when compared to our previous work on graphene-based lasers^25^. This is caused by a combination of several factors. The CNTs seem to have a higher saturation threshold, so it was required to pump the laser significantly stronger. We have also used a different active fiber with higher gain (a new, improved version of the Tm-doped fiber from Nufern), and higher quality components, which introduce smaller losses. This resulted in significantly increased conversion efficiency of the lasers.

**Figure 4:**
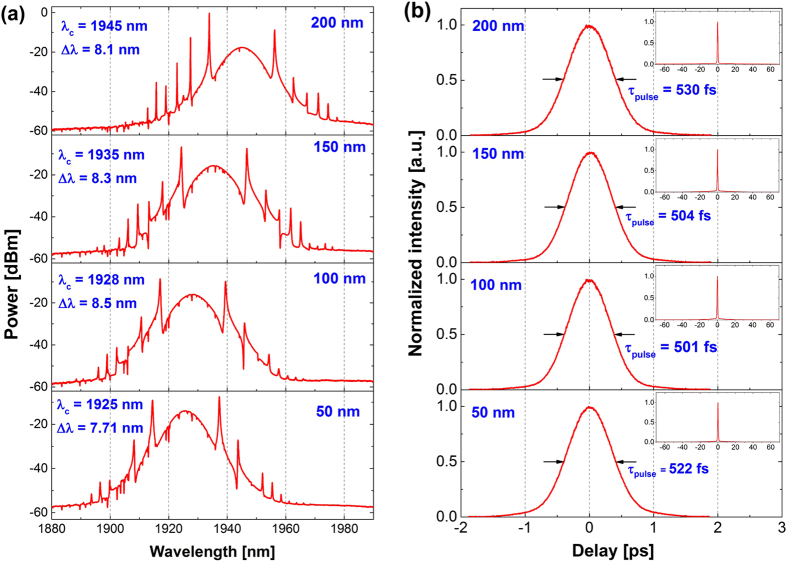
Recorded optical spectra (**a**) and pulse autocorrelation traces (**b**) for different CNT layer thickness in the saturable absorber.

Figure 4 Shows the measured optical spectra for all four cases (**a**) and the corresponding autocorrelation traces (**b**). The spectrum red-shifts with the increasing thickness of the CNT layer due to increased attenuation of the SA. The autocorrelations measured in a wide scanning range (140 ps) are depicted as inset graphs in Fig. 4(b). It can be seen, that the AC traces are free of any pre- or post-pulses, or any signs of harmonic mode-locking, Q-switching, etc.

**Figure 5:**
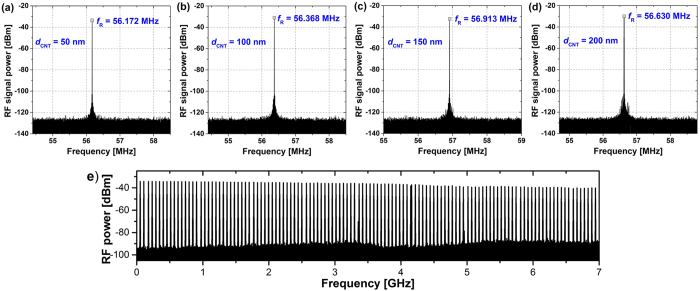
Measured RF spectra of the lasers with different CNT layer thickness in the saturable absorber: 50 nm (**a**), 100 nm (**b**), 150 nm (**c**), 200 nm (**d**), exemplary RF spectrum measured with 7 GHz span using 100 nm CNT layer (**e**).

The measured radio frequency (RF) spectra of the laser with four CNT-SAs are depicted in Fig. 5. The spectra in figures (a–d) are centered at the first harmonic of the RF comb, and confirm that the cavity length was maintained in all oscillators. In all cases the signal to noise ratio (SNR) was at a very high level, exceeding 70 dB. Figure 5(e) depicts an exemplary spectrum recorded in the full available frequency span (taken from the oscillator with 100 nm CNT-SA) showing a clean comb of harmonics without any parasitic modulations or filtering.

## Methods

### Optical characterization of the saturable absorbers

**Figure 6:**
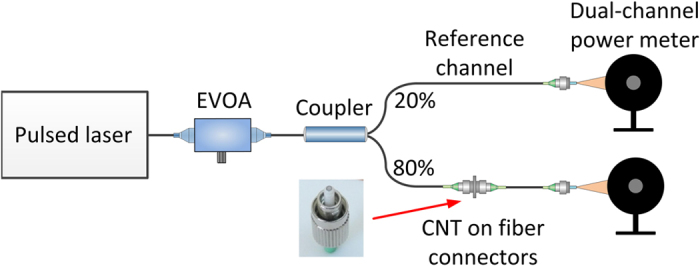
Power-dependent transmission measurement setup.

The saturable absorption curves shown in Fig. 2 were retrieved using an all-fiber power-dependent transmittance setup, depicted in Fig. 6^44^,^45^. A commercially available femtosecond laser was used as pumping source (Menlo Systems T-Light, 100 MHz repetition rate). The laser beam was divided into two parts by a 80/20% coupler. The average power incident on the sample was tuned by an electrically controlled variable optical attenuator (EVOA). The power in both arms measured by the optical power meter (Newport 2936-R with 918D-IG-OD3R heads). The pulse duration at the sample was at the level of 550 fs, and it was adjusted to be similar to the pulse duration achieved from the Tm-doped laser. The maximum average power at the measured sample was 50 mW. The measurement data were fitted with a commonly used formula valid for fast SAs^46^,^47^:


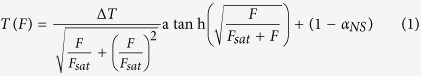


where *ΔT* denotes the modulation depth, *α*_*NS*_ are the non-saturable losses, and *F*_*sat*_ is the saturation fluence.

### Laser characterization

The performance of the laser in all configurations was observed using an optical spectrum analyzer with scanning range up to 2400 nm (Yokogawa AQ6375), an RF spectrum analyzer (Agilent EXA N9010A) with 7 GHz bandwidth, coupled with a 16 GHz photodetector (Discovery Semiconductors DSC2-50S), an optical autocorrelator (Femtochrome FR-103XL), and optical power meter (Gentec Maestro with XLP12-3S-VP detector).

### CNT film characterization

SEM images of the CNT films were performed with using Raith e_Line PLUS microscope. The SEM images of all our samples looks very similar, therefore only two extreme thicknesses are shown in Fig. 1. More can be found in our previous work on CNT film fabrication (ref. 39). For the transmittance measurements as a function of the wavelength a Theremo Scientific Nicolet iS50 spectrometer was used. The Raman spectra were collected using a Renishaw spectrometer with a 514 nm laser excitation line (2.41 eV) in a back-scattering configuration.

### CNT film fabrication

The CNT thin films were produced using water solution of separated semiconducting single wall carbon nanotubes from Nanointegris (Iso-Nanotubes, 99% purity, mean diameter of 1.4 nm) with the tube concentration of 0.01 mg/ml. An appropriate amount of solution was vacuum filtered^26^ onto the Mixed Cellulose Ester (MCE) membrane from Millipore (0.025 μm pore size, 25 mm diameter) to achieve a specified thickness of the CNT thin films: 50, 100, 150 and 200 nm. After vacuum filtration process a dry 5 × 5 mm piece of each CNT film coated MCE membrane was immersed in acetone, where the filter was quickly dissolved. The process was repeated through several baths to ensure effectively complete removal of MCE. Then acetone was replaced by the isopropanol/water solution with the ratio of 1:1 and CNT film was picked up on a fiber connector and gently dried by the nitrogen stream. We note that this method allows to produce high-density films with a uniform thickness and, also important, high purity without residual surfactants (proved by Raman and SEM).

## Summary and conclusions

Summarizing, we have presented a study on the performance of a mode-locked Tm-doped fiber laser, depending on the nanotube layer thickness in the saturable absorber. We have fabricated a set of four CNT layers with different thicknesses (ranging from 50 to 200 nm), and deposited them onto fiber connectors forming saturable absorbers for the fiber laser. All samples were characterized in terms of nonlinear optical properties and tested in a state-of-the art, Tm-doped fiber soliton laser. Our experiments have shown, that the best performance in terms of pulse duration is obtained with 100 nm thick nanotube film in the SA. Such thickness provides the best balance between the modulation depth, non-saturable losses and saturation fluence in a typical, low-power TDFL. At this value, the SA exhibits over 13% of modulation depth, which supports generation of 500-fs short optical pulses centered at 1925 nm. We have shown, that further increasing of the CNT thickness does not improve the performance of the laser, most likely because of too high non-saturable losses and insufficient intra-cavity fluence. However, our study has revealed, that with all absorbers the performance of the laser remains at a quite comparable level (pulse duration between 501 and 530 fs). Changing the thickness of the SA mostly influences only the central wavelength of the emission, between 1925 and 1945 nm. Choosing the SA with proper CNT thickness (which is controllable in our process) might be an efficient method for wavelength-tuning of the laser, without degrading the pulse duration, bandwidth, or output power.

## Additional Information

**How to cite this article:** Sobon, G. *et al*. CNT-based saturable absorbers with scalable modulation depth for Thulium-doped fiber lasers operating at 1.9 µm. *Sci. Rep.* **7**, 45491; doi: 10.1038/srep45491 (2017).

**Publisher's note:** Springer Nature remains neutral with regard to jurisdictional claims in published maps and institutional affiliations.

## Figures and Tables

**Table 1:** Summary of the obtained nonlinear parameters of the saturable absorbers.

CNT thickness [nm]	Saturation fluence (*F*_*sat*_) [μJ/cm^2^]		Modulation depth (*ΔT*) [%]	
	Measured	Used for fitting	Measured	Used for fitting
50	53	100	3.1	4.9
100	61	120	8.1	13.3
150	80	180	8.6	15.9
200	71	180	9.1	17.3

**Table 2:** Summary of the obtained mode-locking parameters.

d_CNT_ [nm]	P_pump_thr_ [mW]	P_pump_max_ [mW]	λ_center_ [nm]	Δλ_FWHM_ [nm]	τ_pulse_ [fs]	P_out_ [mW]	E_p_ [nJ]	TBP	f_rep_ [MHz]
50	570	620	1925.8	7.7	522	22	0.39	0.325	56.172
100	410	455	1928.5	8.5	501	28.5	0.50	0.343	56.368
150	478	538	1935.5	8.3	504	27.5	0.48	0.335	56.913
200	444	530	1945.1	8.1	530	35	0.62	0.340	56.630

## References

[b1] Fried N. M. & Murray K. E. High-power thulium fiber laser ablation of urinary tissues at 1.94 μm. J. Endourol. 19, 25–31 (2005). 15735378 10.1089/end.2005.19.25

[b2] Szlauer R., Götschl R., Razmaria A., Paras L. & Schmeller N. T. Endoscopic vaporesection of the prostate using the continuous-wave 2-micron thulium laser: outcome and demonstration of the surgical technique. Eur. Urol. 55, 368–375 (2009). 19022557 10.1016/j.eururo.2008.10.034

[b3] Hüttmann G., Yao C. & Endl E. New concepts in laser medicine: Towards a laser surgery with cellular precision. Med. Las. Appl. 20, 135–139 (2005).

[b4] Stark A. . Intracavity absorption spectroscopy with thulium-doped fibre laser. Opt. Commun. 215, 113–123 (2003).

[b5] Khodabakhsh A. . Fourier transform and Vernier spectroscopy using an optical frequency comb at 3–5.4 μm. Opt. Lett. 41, 2541–2544 (2016). 27244409 10.1364/OL.41.002541

[b6] Kubat I. . Thulium pumped mid-infrared 0.9–9 μm supercontinuum generation in concatenated fluoride and chalcogenide glass fibers. Opt. Express 22, 3959–3967 (2014). 24663717 10.1364/OE.22.003959

[b7] Luo J. . Mid-IR supercontinuum pumped by femtosecond pulses from thulium doped all-fiber amplifier. Opt. Express 24, 13939–13945 (2016). 27410556 10.1364/OE.24.013939

[b8] Wang Q., Geng J., Luo T. & Jiang S. Mode-locked 2 μm laser with highly thulium-doped silicate fiber. Opt. Lett. 34, 3616–3618 (2009). 19953138 10.1364/OL.34.003616

[b9] Solodyankin M. A. . Mode-locked 1.93 μm thulium fiber laser with a carbon nanotube absorber. Opt. Lett. 33, 1336–1338 (2008). 18552950 10.1364/ol.33.001336

[b10] Kieu K. & Wise F. W. Soliton thulium-doped fiber laser with carbon nanotube saturable absorber. IEEE Photon. Technol. Lett. 21, 128–130 (2009). 10.1109/LPT.2008.2008727 PMC3127443 21731403

[b11] Wang Q. . All-fiber passively mode-locked thulium-doped fiber ring laser using optically deposited graphene saturable absorbers. Appl. Phys. Lett. 102, 131117 (2013).

[b12] Fu B. . Broadband Graphene Saturable Absorber for Pulsed Fiber Lasers at 1, 1.5, and 2 μm. IEEE J. Sel. Top. Quantum Electron. 20, 1100705 (2014).

[b13] Jung M. . A femtosecond pulse fiber laser at 1935 nm using a bulk-structured Bi_2_Te_3_ topological insulator. Opt. Express 22, 7865–7874 (2014). 24718162 10.1364/OE.22.007865

[b14] Sotor J. . Ultrafast thulium-doped fiber laser mode locked with black phosphorus. Opt. Lett. 40, 3885–3888 (2015). 26274685 10.1364/OL.40.003885

[b15] Li J. . All-fiber passively mode-locked Tm-doped NOLM-based oscillator operating at 2-μm in both soliton and noisy-pulse regimes. Opt. Express 22, 7875–7882 (2014). 24718163 10.1364/OE.22.007875

[b16] Wang F. . Wideband-tuneable, nanotube mode-locked, fibre laser. Nat. Nanotech. 3, 738–742 (2008). 10.1038/nnano.2008.312 19057594

[b17] Hasan T. . Nanotube–Polymer Composites for Ultrafast Photonics. Adv. Mater. 21, 3874–3899 (2009).

[b18] Zhang H., Tang D., Zhao L., Bao Q. & Loh K. Vector dissipative solitons in graphene mode locked fiber lasers. *Opt*. Commun. 283, 3334–3338 (2010).

[b19] Yu Z. . A 66 fs highly stable single wall carbon nanotube mode locked fiber laser. Laser Phys. 24, 015105 (2014).

[b20] Ono T. . A 31 mW, 280 fs passively mode-locked fiber soliton laser using a high heat-resistant SWNT/P3HT saturable absorber coated with siloxane. Opt. Express 20, 23659–23665 (2012). 23188331 10.1364/OE.20.023659

[b21] Wang J. . 152 fs nanotube-mode-locked thulium-doped all-fiber laser. Sci. Rep. 6, 28885 (2016). 27374764 10.1038/srep28885 PMC4931500

[b22] Chernysheva M. A. . Thulium-doped mode-locked all-fiber laser based on NALM and carbon nanotube saturable absorber. Opt Express 20, B124–30 (2012). 23262842 10.1364/OE.20.00B124

[b23] Chernysheva M. A. . SESAM and SWCNT mode-locked all-fiber thulium-doped lasers based on the nonlinear amplifying loop mirror. IEEE J. Sel. Top. Quantum. Electron. 20, 1101208 (2014).

[b24] Chernysheva M. A. . Higher-order soliton generation in hybrid mode-locked thulium-doped fiber ring laser. IEEE J. Sel. Top. Quantum. Electron. 20, 1100908 (2014).

[b25] Sobon G. . Multilayer graphene-based saturable absorbers with scalable modulation depth for mode-locked Er- and Tm-doped fiber lasers. Opt. Mater. Express 5, 2884–2894 (2015).

[b26] Wu Z. . Transparent, conductive carbon nanotube films. Science 305, 1273–76 (2004). 15333836 10.1126/science.1101243

[b27] Duzynska A., Taube A., Korona K. P., Judek J. & Zdrojek M. Temperature-dependent thermal properties of single-walled carbon nanotube thin films. Appl. Phys. Lett. 106, 183108 (2015).

[b28] Liu X. & Cui Y. Flexible pulse-controlled fiber laser. Sci. Rep. 5, 9399 (2015). 25801546 10.1038/srep09399 PMC4371082

[b29] Liu X. . Versatile multi-wavelength ultrafast fiber laser mode-locked by carbon nanotubes. Sci. Rep. 3, 2718 (2013). 24056500 10.1038/srep02718 PMC3779847

[b30] Cui Y. & Liu X. Graphene and nanotube mode-locked fiber laser emitting dissipative and conventional solitons. Opt. Express 21, 18969–18974 (2013). 23938811 10.1364/OE.21.018969

[b31] Kobtsev S. . Ultrafast all-fibre laser mode-locked by polymer-free carbon nanotube film. Opt. Express 24, 28768–28773 (2016). 27958520 10.1364/OE.24.028768

[b32] Jeong H. . All-fiber mode-locked laser oscillator with pulse energy of 34 nJ using a single-walled carbon nanotube saturable absorber. Opt. Express 22, 22667–22672 (2014). 25321735 10.1364/OE.22.022667

[b33] Choi S. Y., Jeong H., Hong B. H., Rotermund F. & Yeom D.-I. All-fiber dissipative soliton laser with 10.2 nJ pulse energy using an evanescent field interaction with graphene saturable absorber. Laser Phys. Lett. 11, 015101 (2014).

[b34] Boguslawski J. . Mode-locked Er-doped fiber laser based on liquid phase exfoliated Sb2Te3 topological insulator. Laser Phys. 24, 105111, 2014.

[b35] Cui Y., Lu F. & Liu X. MoS2-clad microfibre laser delivering conventional, dispersion-managed and dissipative solitons. Sci. Rep. 6, 30524 (2016). 27456468 10.1038/srep30524 PMC4960607

[b36] Liu X. M., Han X. X. & Yao X. K. Discrete bisoliton fiber laser. Sci. Rep. 6, 34414 (2016). 27767075 10.1038/srep34414 PMC5073350

[b37] Zhang H., Tang D., Zhao L., Bao Q. & Loh K. P. Vector dissipative solitons in graphene mode locked fiber lasers. Opt. Commun. 283, 3334–3338 (2010).

[b38] Zhang H., Tang D. Y., Zhao L. M. & Xiang N. Coherent energy exchange between components of a vector soliton in fiber lasers. Opt. Express 16, 12618–12623 (2008). 18711498 10.1364/oe.16.012618

[b39] Duzynska A. . Phonon properties in different types of single walled carbon nanotube thin films probed by Raman spectroscopy. Carbon 105, 377–386 (2016).

[b40] Orsila L., Härkönen A., Hyyti J., Guina M. & Steinmeyer G. Ultrahigh precision nonlinear reflectivity measurement system for saturable absorber mirrors with self-referenced fluence characterization. Opt. Lett. 39, 4384–4387 (2014). 25078183 10.1364/OL.39.004384

[b41] Popa D. . 74-fs nanotube-mode-locked fiber laser. Appl. Phys. Lett. 101, 153107 (2012).

[b42] Cho W. B. . Boosting the Nonlinear Optical Response of Carbon Nanotube Saturable Absorbers for Broadband Mode-Locking of Bulk Lasers. Adv. Funct. Mater. 20, 1937–1943 (2010).

[b43] Choi S. Y. . Octave Spanning Ultra-Broadband Carbon Nanotube Saturable Absorber for Bulk Solid-State Lasers. In: Advances in Optical Materials, OSA Technical Digest (Optical Society of America, 2011), paper AWB4.

[b44] Sobon G. Mode-locking of fiber lasers using novel two-dimensional nanomaterials: graphene and topological insulators [Invited]. Photon. Res. 3, A56–A63 (2015).

[b45] Chernysheva M. . Carbon nanotubes for ultrafast fibre lasers. Nanophotonics, doi: 10.1515/nanoph-2015-0156 (2016).

[b46] Zaugg C. . Ultrafast and widely tuneable vertical-external-cavity surface-emitting laser, mode-locked by a graphene-integrated distributed Bragg reflector. Opt. Express 21, 31548–31559 (2013). 24514728 10.1364/OE.21.031548

[b47] Schibli T., Thoen E., Kärtner F. & Ippen E. Suppression of Q-switched mode locking and break-up into multiple pulses by inverse saturable absorption. Appl. Phys. B 70, S41–S49 (2000).

